# Clinicopathological characteristics and survival in colorectal signet ring cell carcinoma: a population-based study

**DOI:** 10.1038/s41598-020-67388-6

**Published:** 2020-06-26

**Authors:** Luo-luo Yang, Min Wang, Ping He

**Affiliations:** 1 Department of Gastroenterology, First Hospital of Jilin University, Changchun, 130021 Jilin China; 2 Department of Pathology, Jilin Province Tumor Hospital, Changchun, 130012 Jilin China

**Keywords:** Cancer, Gastroenterology, Oncology, Risk factors

## Abstract

We aimed to reveal clinicopathological features and explore survival-related factors of colorectal signet ring cell carcinoma (SRCC). A population-based study was carried out to investigate colorectal SRCC by using data extracted from the surveillance, epidemiology and end results (SEER) database between 2004 and 2015. In total, 3,278 patients with colorectal SRCC were identified, with a median age of 63 (12–103) years old. The lesions of most patients (60.49%) were located in the cecum–transverse colon. In addition, 81.27% patients had advanced clinical stage (stage III/IV), and 76.69% patients had high pathological grade. The 3–, 5–year cancer‐specific survival and overall survival rate was 35.76%, 29.32% and 32.32%, 25.14%. Multivariate analysis revealed that primary site in cecum–transverse colon, married, received surgery, lymph node dissections ≥ 4 regional lymph nodes were independent favorable prognostic. Meanwhile, aged ≥ 65 years, higher grade, tumor size ˃5 cm and advanced AJCC stage were associated with poor prognosis. Patient age, tumor grade, marital status, tumor size, primary tumor location, AJCC stage, surgery and number of dissected lymph node had significant correlation with prognosis of colorectal SRCC.

## Introduction

Colorectal cancer (CRC) is still a main global health burden, ranking the second of all types of malignancies in terms of cancer-associated mortality^1^. Recently accumulative attention has been paid to colorectal signet ring cell carcinoma (SRCC), initially proposed by Saphir and Laufman in 1951^2^. Stomach is considered as the most common site for primary SRCC, while colorectal SRCC is less frequent^3^. In addition, colorectal SRCC is a very rare and special type of all CRCs, which has its unique clinicopathological characteristics and prognosis^4-8^.

Various researches have shown the origination of SRCC from undifferentiated stem cells of colorectal mucosa, thus, rapid growth, poor differentiation, diffuse infiltration as well as high metastatic rate are generally observed^9,10^. Moreover, SRCC has been considered as an independent prognostic indicator for poor outcome based on the AJCC stage system^11^. However, the currently limited understanding of colorectal SRCC is mainly based on case reports as well as small case series, without complete elucidation on the clinicopathological features and prognosis^12^.

The National Institutes of Health (NIH)’ s Surveillance, Epidemiology and End Results (SEER) database, the most authoritative and largest cancer dataset in North America^13^, covers nearly 30% of population in the USA from different geographic regions^14^, which provides valuable data to investigate rare malignancies^15-17^. Therefore, we performed a retrospective analysis of patients with colorectal SRCC registered in the SEER database to summarize clinical characteristics and survival and to delineate features influencing prognosis.

## Materials and methods

### Ethics statement

In order to obtain relevant data from the database, we signed the SEER Research Data Agreement (No. 19817-Nov2018) and further searched for data according to the approved guidelines. The extracted data were publicly available and de-identified, and the data analysis was considered as non-human subjects by Office for Human Research Protection, therefore, no approval was required from institutional review board.

### Study population

SEER*State v8.3.6 tool was used to select qualified subjects. Colorectal SRCC patients who were diagnosed from January 1, 2004 to December 31, 2015 were selected from the Incidence-SEER 18 Registries Custom Data (with additional treatment fields), released August 2019, based on the November 2018 submission. The inclusion criteria included the following: (1) it should be primary colorectal SRCC patients; (2) SRCC diagnosed in line with the International Classification of Disease for Oncology, Third Edition (ICD-O-3; coded as 8,490/3). The exclusion criteria were listed in the following: (1) Patients had multiple primary tumors; (2) Patients with reported diagnosis source from autopsy or death certificate or only clinically diagnosed; (3) Patients did not have some important clinicopathological information, including: AJCC stage and surgical style; (4) Patients had no prognostic data. The remaining qualified populations were included.

### Covariates and endpoint

We analyzed the patients’ characteristics according to the following factors: year of diagnosis (2004–2007, 2008–2011, 2012–2015); insured status (uninsured/unknown, any medicaid/insured; age(< 65,  ≥ 65); marital status (unmarried, married); gender (female, male); race (black, white or others); primary site(cecum–transverse colon, descending colon–sigmoid, multiple, rectum and unknown); grade (grade I/II, grade III/IV, unknown); tumor size (≤ 5 cm, ˃5 cm,unknown); AJCC stage ( stage I, II, III, IV); surgery(no surgery, local tumor excision /partial colectomy, total colectomy), and lymph node dissection (none or biopsy, 1 to 3 regional lymph nodes removed, ≥ 4 regional lymph nodes removed, unknown).The widowed or single (never married or having a domestic partner) or divorced or separated patients were classified as unmarried. The primary tumor site was classified as cecum–transverse colon (including the cecum, appendix, ascending colon, hepatic flexure and the transverse colon), descending colon–sigmoid (including the splenic flexure and descending and sigmoid colons), multiple, rectum and unknown^18^. Year of diagnosis was equally divided into 2004–2007, 2008–2011, 2012–2015, which was referred to the previous papers^19,20^. The grouping of the age^21^ and tumor size^22^ also refers to the published studies. In addition, the staging of cancer is based on the 6th edition of AJCC stage system, which adapted to patients in the SEER database with a diagnosis time of 2004–2015^23^.

The endpoint of this study was cancer‐specific survival (CSS) and overall survival (OS). CSS was defined as the period from diagnosis to death attributed to colorectal SRCC. OS was defined as the period from diagnosis to death from any cause.

### Statistical analysis

Kaplan–Meier (K–M) method was employed for univariate analysis and stratified analysis, followed by log-rank test to determine the differences of OS and CSS. Of note, if variables had *P* values ≤ 0.1 in univariate analysis, they were incorporated into multivariate Cox proportional hazard analysis. SPSS software version 19.0 (SPSS Inc., Chicago, USA) was adopted for statistical analysis, and GraphPad Prism 5 was adopted for plotting survival curves. A two-sided *P* < 0.05 was suggestive of statistical significance.

## Results

### Patients’ characteristics

**Figure 1:**
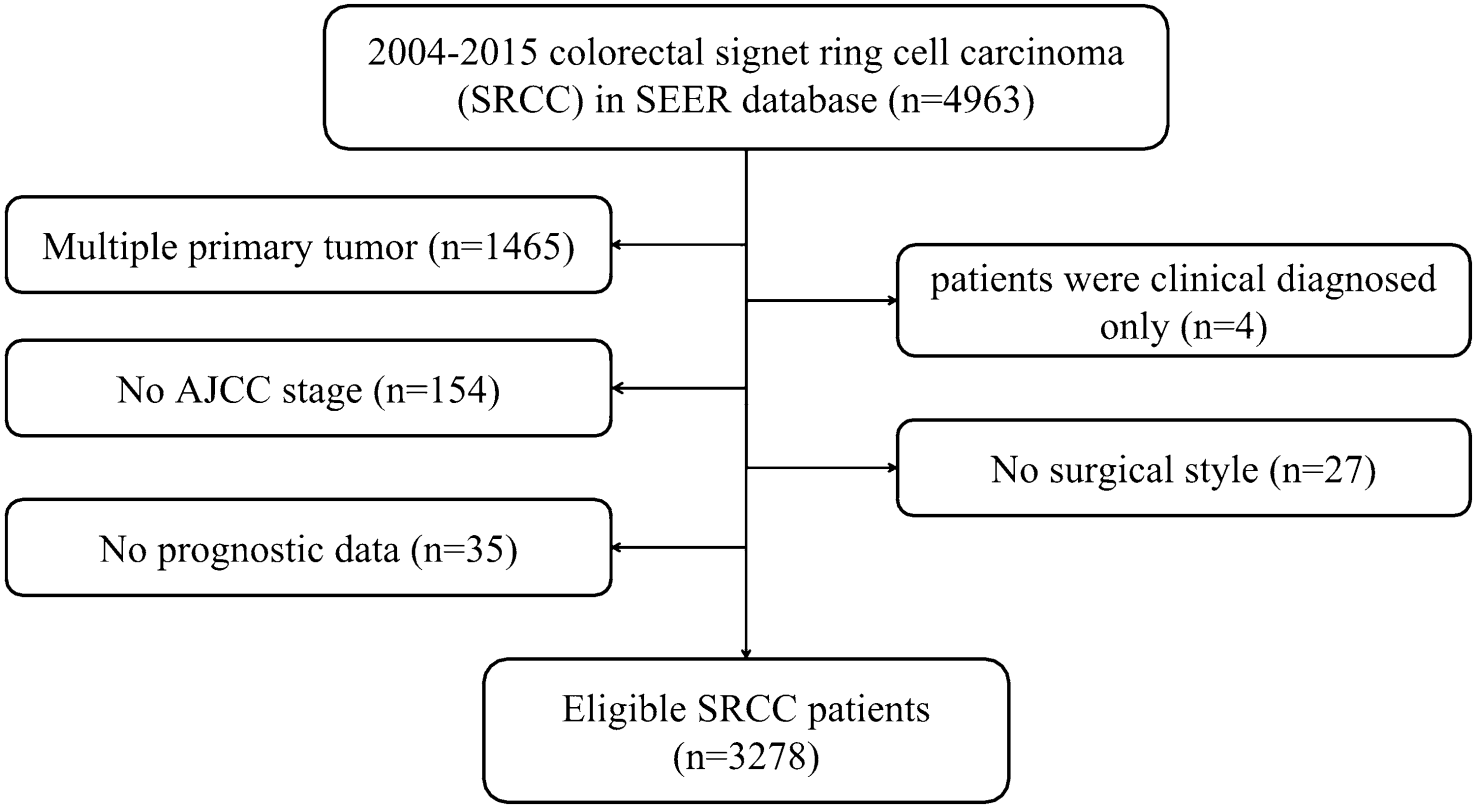
Flow chart of patient selection.

Based on the inclusion and exclusion criteria, 3,278 eligible subjects were included. The flowchart of patient selection was displayed in Fig. 1.The patients’ features and therapeutic regimens were listed in Table 1. Of all the included patients, the median age was 63 (12–103) years old, with the male to female ratio of nearly 1:1.The lesions were mostly located in cecum–transverse colon (60.49%), 640 cases (19.52%) were detected in rectum, 515 cases (15.71%) were observed in descending colon–sigmoid and only 50 cases (1.53%) were located in overlapping of colon. Most of colorectal SRCC were in advanced clinical stage (stage III/IV: 81.27%) and high pathological grade (grade III/IV: 76.69%). Surgical resection was performed on 2,602 (79.38%) patients, including 1738 (53.02%) patients received total colectomy. Most patients (70.41%) had ≥ 4 regional lymph nodes removed.

**Table 1:** The clinicopathological characteristics and treatment of the included 3,278 colorectal signet ring cell carcinoma patients.

Variable	N (%)
**Year at diagnosis**	
2004–2007	1,098 (33.50%)
2008–2011	1,066 (32.52%)
2012–2015	1,114 (33.98%)
**Insured status**	
uninsured/unknown	1,017 (31.03%)
any medicaid/insured	2,261 (68.97%)
**Age**	
< 65	1,741 (53.11%)
≥ 65	1,537 (46.89%)
**Marital status**	
Unmarried	1,501 (45.79%)
Married	1,777 (54.21%)
**Gender**	
Female	1,601 (48.84%)
Male	1,677 (51.16%)
**Race**	
Black	312 (9.52%)
White	2,685 (81.91%)
Other	281 (8.57%)
**Primary site**	
Cecum–transverse colon	1,983 (60.49%)
Descending colon–sigmoid	515 (15.71%)
Multiple	50 (1.53%)
Rectum	640 (19.52%)
Unknown	90 (2.75%)
**Grade**	
Grade I/II	176 (5.37%)
Grade III/IV	2,514 (76.69%)
Unknown	588 (17.94%)
**Tumor size**	
≤ 5 cm	1,219 (37.19%)
> 5 cm	1,267 (38.65%)
Unknown	792 (24.16%)
**AJCC stage**	
I	154 (4.70%)
II	460 (14.03%)
III	1,215 (37.07%)
IV	1,449 (44.20%)
**Surgery**	
No surgery	676 (20.62%)
Local tumor excision/partial colectomy	864 (26.36%)
Total colectomy	1,738 (53.02%)
**Lymph node dissection**	
None or biopsy	884 (26.97%)
1—3	86 (2.62%)
≥ 4	2,308 (70.41%)

### Patient survival

**Figure 2:**
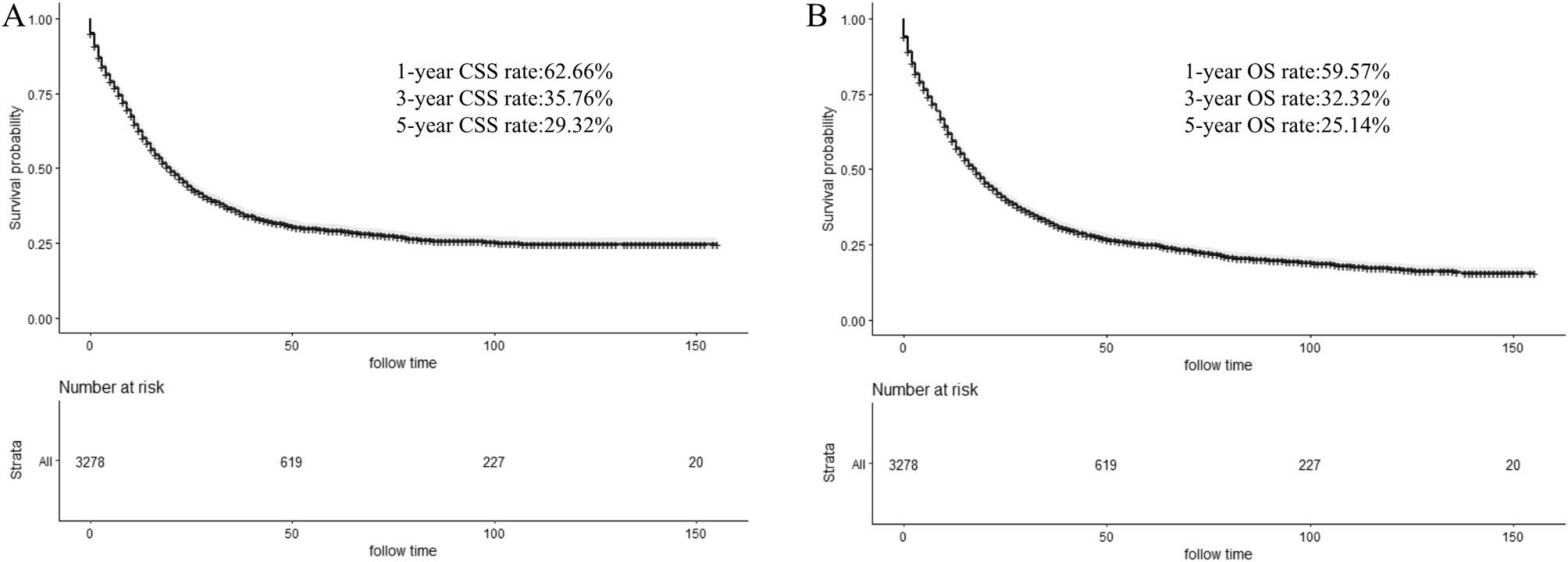
Kaplan–Meier curves for cancer-specific survival (CSS) (**A**) and overall survival (OS) (**B**) of included patients.

**Figure 3:**
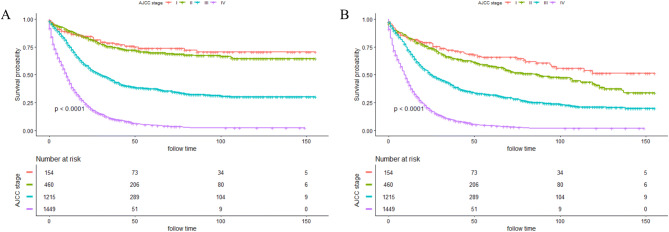
Kaplan–Meier curves stratified by AJCC stage of CSS (**A**) and OS (**B**).

The median survival time of all included patients was 16.0 months (0–155 months). The 1–, 3– and 5–year CSS rate was 62.66%, 35.76%, and 29.32%, respectively (Fig. 2A). Meanwhile, the 1–, 3–and 5–year OS rate was 59.57%, 32.32% and 25.14%, respectively (Fig. 2B). K-M curves based on AJCC stage was displayed in Fig. 3A (CSS) and Fig. 3B (OS). Significantly poorer prognosis was detected in subjects with stages III or IV compared to those with stages I or II (both *P* < 0.0001). The 5–year CSS and OS rate in subjects with stage I were 73.66% and 65.63%; stage II: 69.81% and 57.72%; stage III: 37.31% and 31.94%; stage IV: 4.80% and 4.24%, indicating inferior outcome in those with stages III or IV.

### Prognostic factors of survival and stratified analysis

In the univariate analyses, marital status, primary tumor location, tumor size, AJCC stage, grade, surgery and dissected lymph node were predictors of CSS (all *P* < 0.05). Additionally, multivariate analysis further demonstrated that married status (HR 0.863, 95% CI 0.792–0.940, *P* = 0.001); primary site in cecum–transverse colon (*P* = 0.001) and surgery [(local tumor excision/partial colectomy) HR 0.609, 95% CI 0.506–0.734; (total colectomy) HR 0.693, 95% CI 0.571–0.840, both *P* < 0.001] were independent prognostic indicators for better survival. Meanwhile, higher grade (HR 1.679, 95% CI 1.347–2.094, *P* < 0.001), tumor size ˃5 cm (HR 1.297, 95% CI 1.170–1.438, *P* < 0.001) and advanced AJCC stage [(stage III) HR 3.353, 95% CI 2.401–4.682; (stage IV) HR 7.723, 95% CI 5.539–10.769, both *P* < 0.001] could independently predict worse prognosis. Similar outcomes were observed in multivariate analysis on OS. Besides, it found that aged ≥ 65 years (HR 1.712, 95% CI 1.574–1.863, *P* < 0.001) and lymph node dissection ≥ 4 (HR 0.777, 95% CI 0.659–0.917, *P* = 0.003) were also independent prognostic indicators of OS (Table 2).

**Table 2:** Univariate and multivariate analyses of cancer special survival (CSS) and overall survival (OS) for 3,278 patients with colorectal SRCC.

Variables	CSS			OS		
	Univariate analysis	Multivariate analysis		Univariate analysis	Multivariate analysis	
	*P* value	HR (95%CI)	*P* value	*P* value	HR (95%CI)	*P* value
Year at diagnosis	0.713		NI	0.865		NI
2004–2007						
2008–2011						
2012–2015						
Insured status	0.599		NI	0.760		NI
Uninsured/unknown						
Any medicaid/insured						
Age	0.731		NI	< 0.001		< 0.001
< 65					Reference	
≥ 65					1.712 (1.574,1.863)	
Marital status	0.003		0.001	< 0.001		0.001
Unmarried		Reference			Reference	
Married		0.863 (0.792,0.940)			0.870 (0.802,0.943)	
Gender	0.148		NI	0.440		NI
Female						
Male						
Race	0.232		NI	0.254		NI
Black						
White						
Other						
Primary site	< 0.001		0.001	< 0.001		< 0.001
Cecum–transverse colon		Reference			Reference	
Descending colon–sigmoid		1.137 (1.004.1.287)	0.043		1.179 (1.048,1.327)	0.006
Multiple		1.431 (1.034,1.980)	0.031		1.327 (0.967,1.821)	0.080
Rectum		1.182 (1.053,1.328)	0.005		1.237 (1.107.1.383)	< 0.001
Unknown		1.397 (1.103,1.768)	0.005		1.359 (1.081,1.708)	0.009
Grade	< 0.001		< 0.001	< 0.001		< 0.001
Grade I/II		Reference			Reference	
Grade III/IV		1.679 (1.347,2.094)	< 0.001		1.672 (1.369,2.042)	< 0.001
Unknown		1.629 (1.282,2.069)	< 0.001		1.618 (1.300,2.013)	< 0.001
Tumor size	< 0.001		< 0.001	< 0.001		< 0.001
≤ 5 cm		Reference			Reference	
> 5 cm		1.297 (1.170,1.438)	< 0.001		1.251 (1.137,1.375)	< 0.001
Unknown		1.276 (1.124,1.448)	< 0.001		1.299 (1.151,1.466)	< 0.001
AJCC stage	< 0.001		< 0.001	< 0.001		< 0.001
I		Reference			Reference	
II		1.210 (0.836,1.751)	0.313		1.387 (1.033,1.864)	0.030
III		3.353 (2.401,4.682)	< 0.001		2.917 (2.221,3.832)	< 0.001
IV		7.723 (5.539,10.769)	< 0.001		6.638 (5.051,8.725)	< 0.001
Surgery	< 0.001		< 0.001	< 0.001		< 0.001
No surgery		Reference			Reference	
Local tumor excision/partial colectomy		0.609 (0.506,0.734)	< 0.001		0.640 (0.536,0.764)	< 0.001
Total colectomy		0.693 (0.571,0.840)	< 0.001		0.744 (0.619,0.893)	0.002
Dissected lymph node	< 0.001		0.059	< 0.001		0.011
None or biopsy		Reference			Reference	
1—3		0.798 (0.594,1.071)	0.133		0.810 (0.615,1.068)	0.136
≥ 4		0.813 (0.682,0.969)	0.020		0.777 (0.659, 0.917)	0.003

CSS: cancer‐specific survival; OS: overall survival; NI, not included in the multivariate survival analysis.

## Discussion

According to the WHO definition, colorectal SRCC is a special type of CRC with prominent intracytoplasmic mucin in over 50% of tumor cells^24^, featured by unique clinical manifestation as well as distinct outcome. Colorectal SRCC has been validated to have poorer prognosis compared with other common CRC^4^. Colorectal SRCC, a rare subtype of CRC, consists of 0.1% to 2.6% of all CRC cases^25,26^. Previous research found similar incidence of SRCC between female and male^6,27^ and most SRCC cases are located in the right colon, with rectal SRCC of approximately 20%^28^. Additionally, the initial diagnosis of SRCC is generally at advanced stage, compared with colorectal non-SRCC^4^. For instance, according to a large cohort study in the USA, 80% of colorectal SRCC were initially diagnosed with stage III and IV, which were only 50% in non-SRCC (*P* < 0.01)^28^. These findings are consistent with our study. Moreover, we found that the median age was 63 years old in patients with colorectal SRCC, and 1537 (46.89%) patients were old patients. Our results should be more reliable in this large population-based study while most previous researches on colorectal SRCC were only based on single institution.

Colorectal SRCC is independently related to increased mortality risks^18,20^. Ramya et al. have revealed that the median OS was 18.6 months by involving 206 subjects with colonic SRCC^7^. In addition, the stage-specific 5-year survival rate was declined with advanced stage. The above results are basically consistent with our results, while the median survival time of our study is shorter (16.0 months).

Multiple studies have evaluated the stage-associated in colorectal SRCC, with consistent outcomes indicating better survival in early-stage SRCC^7^. Tumor location may be another independent prognostic indicator^29^. These two prognostic factors are both found in our study. In addition, we found that pathological grade and tumor size were independent prognostic factors of colorectal SRCC.

In terms of the optimal therapeutic regimens, the multidisciplinary management is required for colorectal SRCC. To be specific, surgical intervention is vitally involved in treating localized tumors^12^. Additionally, a large population-based study has evaluated the significance of adjuvant chemotherapy by extracting relevant data from nationwide population-based Netherlands Cancer Registry, which enrolled 1972 subjects with colorectal SRCC from 1989 to 2010. In this study, better survival outcome was detected in stage III colon SRCC patients receiving adjuvant chemotherapy than those without^6^. Tao Shi et al. also found an association between chemotherapy and better survival outcomes in colorectal SRCC patients suffering from distant metastasis^21^. In contrast, the role of radiotherapy in colorectal SRCC is less studied which has not been evaluated alone or combined with chemotherapy in SRCC histology to date. Nevertheless, neoadjuvant chemoradiotherapy could give rise to good therapeutic response in rectal SRCC population^30^. This study further emphasized the importance of operation in colorectal SRCC, including the number of dissected lymph node, and regional lymph node dissection ≥ 4 significantly improved the prognosis of patients.

To our knowledge, the current research is the largest population-based study to explore prognostic indicators in colorectal SRCC in the past decade. In total, 3,278 colorectal SRCC patients were analyzed after retrieving from SEER database. SEER database renders the assessment of a large cross-section of cancer patients and provides long-term follow-up information without inherent institutional biases. Meanwhile, there are certain limitations in our research. Firstly, as a retrospective research based on SEER database, the intrinsic selection biases exists in this study^15,17^. Furthermore, not all data are available from the SEER database, such as molecular-genetic profiles. Thirdly, as the radiotherapy and chemotherapy variables have a relatively low sensitivity, we didn't include the data of radiotherapy and chemotherapy to avoid reporting misleading results. Finally, we could not determine the therapeutic response or recurrence rate according to the data extracted from SEER. Thus, further investigations are warranted in the future.

## Conclusion

In conclusion, we analyzed clinicopathological features as well as survival of colorectal SRCC patients using a population-based approach. Consequently, patient age, tumor grade, marital status, tumor size, primary tumor location, AJCC stage, surgery and dissected lymph node had significant correlation with prognosis of colorectal SRCC. Our study is the largest population-based research to investigate clinicopathological features and survival of patients with colorectal SRCC. Hopefully, our findings are of great significance for the management and future prospective studies for colorectal SRCC.
